# Intimate partner violence during pregnancy and maternal and child health outcomes: a scoping review of the literature from low-and-middle income countries from 2016 - 2021

**DOI:** 10.1186/s12884-022-04604-3

**Published:** 2022-04-13

**Authors:** Thao Da Thi Tran, Linda Murray, Thang Van Vo

**Affiliations:** 1 School of Health Sciences, College of Health, Massey University, Wellington, Aotearoa New Zealand; 2 Institute for Community Health Research, University of Medicine and Pharmacy, Hue University, Hue City, Vietnam; 3 Faculty of Public Health, University of Medicine and Pharmacy, Hue University, Hue City, Vietnam

## Abstract

**Background:**

Intimate partner violence (IPV) during pregnancy is significantly associated with negative outcomes for both mother and child. Current evidence indicates an association between low levels of social support and IPV, however there is less evidence from low-and-middle income countries (LMIC) than high-income countries. Globally, the COVID-19 pandemic has radically altered how women can access social support. Hence since 2020, studies investigating IPV and pregnancy have occurred within the changing social context of the pandemic.

**Objective:**

This scoping review summarizes the evidence from LMICs about the effects of IPV during pregnancy on maternal and child health. The review includes the impact of the COVID-19 pandemic on social support as mentioned in studies conducted since 2020.

**Design:**

Library databases were used to identify papers from 2016 to 2021. These studies reported the maternal and child health outcomes of IPV during pregnancy, and described how social support during pregnancy, and the COVID-19 pandemic, were associated with rates of IPV during pregnancy. Observational study designs, qualitative and mixed methods studies were included.

**Results:**

Twenty - six studies from 13 LMICs were included. Half (n = 13) were cross sectional studies which only collected data at one time-point. IPV during pregnancy was significantly associated with higher odds of postpartum depression, low birth weight, preterm birth and less breastfeeding in the year after birth. Lower levels of social support increased the odds of experiencing IPV during pregnancy, whilst higher levels of social support reduced antenatal anxiety and depression in women experiencing IPV during pregnancy. Of the four studies that investigated IPV during pregnancy throughout the COVID-19 pandemic, only one compared prevalence before and after the pandemic and unexpectedly reported a lower prevalence.

**Conclusions:**

Further research on the impact of IPV during pregnancy on maternal and child outcomes in LMICs is required, especially evidence from longitudinal studies investigating a wider range of outcomes. To date, there is limited evidence on the impact of the COVID-19 pandemic on IPV during pregnancy in LMICs, and this should be prioritized as the pandemic continues to affect women’s access to social support globally.

**Supplementary information:**

The online version contains supplementary material available at 10.1186/s12884-022-04604-3.

## Background

### Rationale

Intimate partner violence (IPV), the most common form of violence against women [1], is defined as “behaviour by an intimate partner that causes physical, sexual or psychological harm, including acts of physical aggression, sexual coercion, psychological abuse and controlling behaviours” [2]. Globally, it is estimated that nearly one in three women aged between 15 and 49 will suffer physical/sexual IPV at least once in their life [1]. IPV during pregnancy is associated with health consequences for both the mother and the expected child. In a systematic review of mainly high-income countries, longitudinal evidence revealed that exposure to IPV during pregnancy tripled the odds of postpartum depression [3]. In low- and middle-income countries (LMICs), perinatal common mental disorders (depression, anxiety, adjustment and somatic disorders) were more prevalent in women exposed to physical IPV during pregnancy or in the previous 12 months compared to unexposed women [4, 5]. IPV during pregnancy is also an established risk factor for antepartum hemorrhage [6], low birth weight [6, 7], intrauterine growth restriction [8], preterm delivery [6], and overall increased fetal morbidity [9]. Moreover, maternal exposure to IPV during pregnancy increases the level of stress hormones reaching the fetus [10], which may affect the behavioral development of the child [9]. Evidence suggests that the odds of both internalizing and externalizing behavioral problems in children whose mothers were exposed to violence during pregnancy were doubled compared to children whose mothers were not exposed to IPV [11]. Therefore, preventing IPV during pregnancy benefits the wellbeing of both mother and child.

Whilst IPV represents a relationship dynamic that is particularly associated with poor perinatal mental health outcomes [4], other forms of social support are also known to be vitally important to women’s wellbeing during the perinatal period. Globally, social distancing restrictions and lockdowns due to the COVID-19 pandemic have markedly affected family living and working arrangements, and access to face-to-face social support [12]. It is well established that pregnant women who have adequate social support (warm, supportive relationships with their partner, family, friends or significant others) are significantly less likely to experience physical or psychological abuse from their spouses [13]. Conversely, having little or no social support independently increases the odds of IPV during pregnancy [14, 15]. The direction of the relationship is unclear from quantitative evidence, but qualitative studies have established that controlling behaviour from an abusive partner can weaken women’s social networks and increase her social isolation [16, 17]. Emerging evidence since the pandemic begun in 2020 has consistently revealed that stay-at-home orders, interrupted access to support services and economic difficulties have worsened violence against women [18, 19]. However, specific information on violence against women who are/were pregnant during the pandemic is scarce. Therefore, this scoping review sought to re-evaluate the role of social support as a protective factor for perinatal mental health in light of this changing global context.

Before the pandemic (2010), an analysis of prevalence data from 19 countries revealed a higher prevalence of IPV during pregnancy in LMICs compared to high-income countries. Population – based research revealed that the prevalence of IPV during pregnancy was higher in African countries (3.8–13.5%), followed by Latin America countries (4.1–11.1%), then Asia (2–5%), while it was only 1.8% in Denmark and 2% in Australia [20]. In correspondence to this risk, the consequences of IPV during pregnancy for maternal and child health are expected to be more frequent in LMICs. However, since IPV was identified as a risk factor for perinatal common mental disorders in LMICs a systematic review by Fisher et al., [4], there has been no further systematic examination of the impact of IPV during pregnancy on both maternal and child health in these countries. Therefore, the purpose of this scoping review is to synthesize literature on IPV during pregnancy in LMICs from the previous five years (2016–2021), which includes research conducted during the COVID-19 pandemic.

### Objectives

Three research questions informed this scoping review of the literature between 2016 and 2021: (1) What is the impact of IPV during pregnancy on maternal and child health in LMICs?, (2) What is the relationship between social support and IPV during pregnancy in LMICs? and (3) What is the impact of the COVID-19 pandemic on IPV during pregnancy in LMICs?

## Methods

### Protocol

As our main aim was to identify and map the literature available rather than to answer a clinical question or inform practice, a scoping review methodology was considered appropriate [21]. This review was conducted using the Joanna Briggs Institute’s updated methodological guidance for the conduct of scoping reviews [22]. The article search was limited to the past five years (2016–2021) to capture the recent literature. The protocol was revised by a researcher at the School of Health Sciences, College of Health, Massey University, New Zealand. Our study is presented according to the *PRISMA Extension for Scoping Reviews: Checklist and Explanation* (PRISMA-ScR) [23].

### Eligibility criteria

The PCC (population, concept, and context) framework was used to select studies for this scoping review [22]. The ***population*** was defined as pregnant women (any time during pregnancy). The ***concept*** encompassed studies that reported at least one of the key measures of interest:

- IPV during pregnancy.
- Social support (search terms relating to partner support, and social/community support were included in the search string – see supplementary material for further information).
- At least one of the key outcomes of interest (mother’s well – being, birth outcomes, child health and well-being).

The ***context*** was LMICs. As defined by the World Bank, low-income countries are those with a gross national income (GNI) per capita of $1,035 or less while middle income countries have GNI per capita between $1,036 and $12,535 [24]. In addition, it was noted that some eligible search results from 2020 to 2021 mentioned the context of the COVID-19 pandemic.

### Study designs

As randomized controlled trials on IPV are ethically impossible [25], we included the studies with observational study designs (cohort, case control, cross – sectional, case series), as well as qualitative or mixed methods studies. Any study that included participants with a known diagnosis/treatment of mental health issues, overt psychosis or concurrent severe physical health problems was excluded.

### Information sources

The search for relevant studies was conducted both electronically and manually. However, the criteria “low- and middle-income countries” was not used as a search term because many articles documented research taking place in an individual country. Therefore the locations of potentially eligible studies were checked against the list of the low- and middle-income countries provided by the World Bank [24].

An electronic search was conducted based on a comprehensive and reproducible strategy of four biomedical bibliographic databases MEDLINE (via Pubmed), Scopus, Web of Science, and PsychINFO. Searches in Google Scholar and Google were also performed to further capture publications from LMICs (grey literature). In addition, a manual search was conducted by going through the “Similar articles” section of an article or the reference lists of eligible studies.

### Search

The search strategy is presented in the Supplementary material.

### Selection of sources of evidence

The search from the four main bibliographic databases yielded 303 studies while the Google/Google scholar search returned 26, resulting in a total of 329. All results were loaded into the online platform Picoportal (picoportal.org), and duplicates were removed, which resulted in 296 studies ready for screening. The criteria for screening studies were built and agreed upon by all authors prior to the selection process (Supplementary material).

**Fig. 1:**
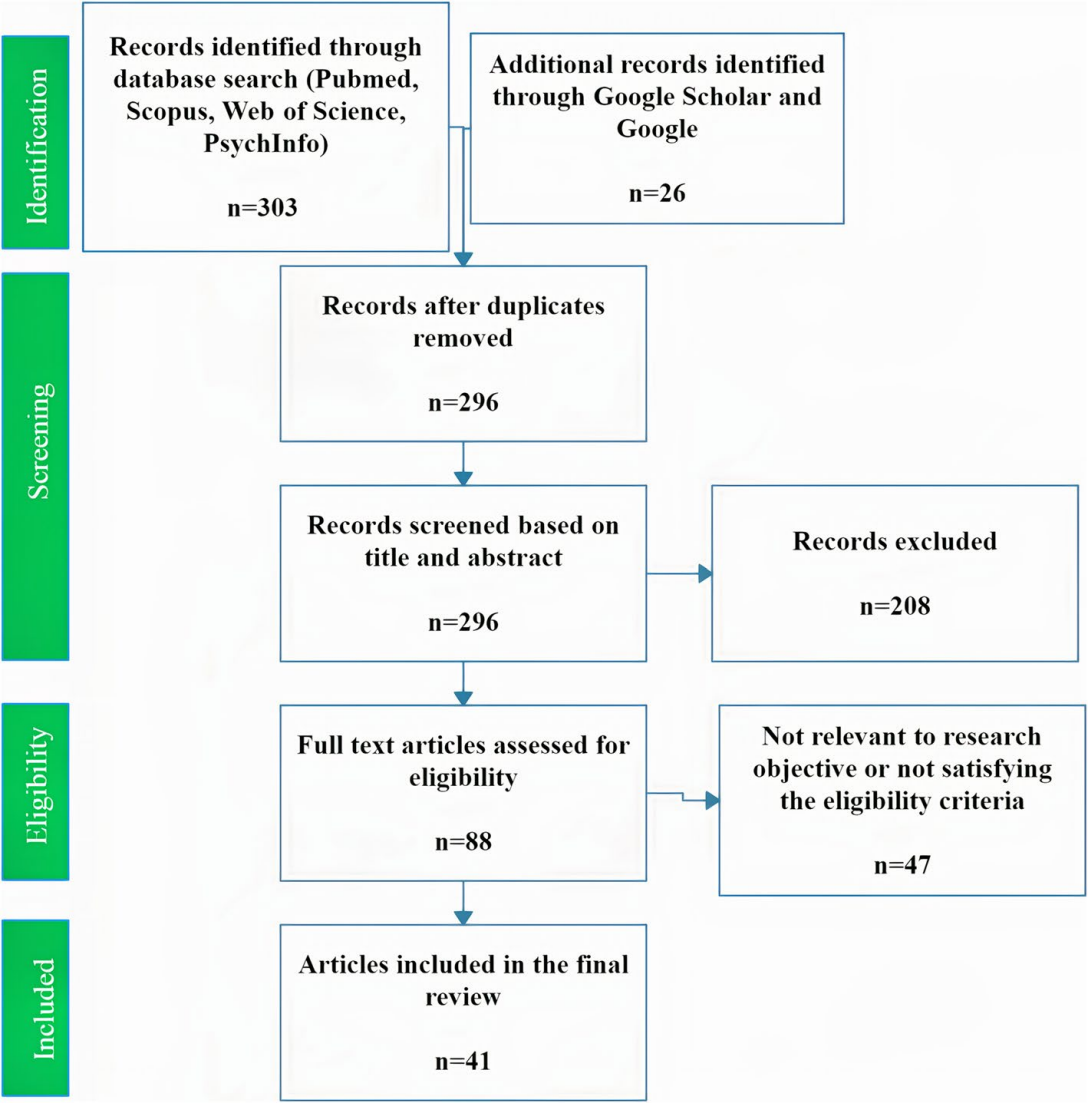
PRISMA flow chart of the study

Two reviewers independently screened the same 296 publications by checking the titles and abstracts. Eighty – eight studies were selected for full text screening. Finally, 26 studies were included in the scoping review. Any disagreement during the screening process was resolved through discussion between the co-authors (Fig. 1).

### Data charting process

Data from the eligible studies were independently extracted by two researchers using the template designed for this study (hosted in freeonlinesurveys.com). The template captured the relevant information on key study characteristics and detailed information on the aforementioned independent and outcome variables.

### Data items

We extracted the data on article characteristics (e.g., title, first author, country of origin), study characteristics (e.g., objective, design, sample), and independent and dependent variables (as defined in the section on eligibility criteria) (the Supplementary material).

It is important to note that IPV during pregnancy is a type of perinatal IPV. Perinatal IPV is defined as the period of 12 months before the pregnancy, during the pregnancy and 12 months after childbirth [26]. As this scoping review focused only on IPV during pregnancy, data in the included studies which described perinatal IPV occurring outside of pregnancy was not included.

### Synthesis of results

The results were grouped by the following themes:

- Evidence about the effect of IPV during pregnancy on maternal and child health.
- Evidence about social support and IPV during pregnancy.
- Evidence about the COVID-19 pandemic and IPV during pregnancy.

## Results

### Characteristics of the included studies

Twenty - six studies were included (characteristics presented in Table 1). Most studies were cross sectional, facility – based, and from African nations, with sample sizes of between 180 and 500. The WHO’s domestic violence questionnaire was the most common tool used to detect IPV (Table 1). The prevalence of IPV during pregnancy was highly variable within and between countries and within research settings (facility/community) (see Supplementary material).

**Table 1:** Characteristics of the 26 included studies

Design	Number of studies
Prospective cohort	5 [27–31]
Cross sectional	13 [8, 32–43]
Quasi – experimental study	1 [44]
Case control	1 [45]
Baseline data of a quasi – experimental study	1 [46]
Secondary analysis of data of a prospective cohort study	1 [47]
Mixed method (quantitative and qualitative)	1 [48]
Qualitative studies	3 [49–51]
**Number of studies using quantitative method**	23 [18–40]
**Number of studies using qualitative method**	4 [48–51]
**Setting (among 23 studies using quantitative method)**	
Facility – based	14 [8, 27, 28, 30, 32, 34, 36–40, 42, 47, 48]
Community – based	9 [29, 31, 33, 35, 41, 43–46]
**Sample size (among 23 studies using quantitative method)**	
180–500	9 [27, 28, 33, 36, 37, 39, 42, 44, 47]
500–1,000	7 [8, 32, 34, 35, 40, 43, 48]
1,000–1,400	6 [29–31, 38, 41, 43]
4,680	1 [46]
**Number of countries represented in this review**	13
**Locations of studies**	
Eastern Africa	Ethiopia [35, 37, 42, 43, 45, 46], Tanzania [38, 51], Uganda [49]
Western Africa	Nigeria [36, 48]
Southern Africa	South Africa [32]
Southern Asia	Iran [28, 34, 39], India [44]
Southeastern Asia	Vietnam [29, 31, 41, 50], Malaysia [40]
Western Asia	Jordan [33]
Between Asia and Europe	Turkey [27]
Latin America	Brazil [8, 30], Mexico [47]
**Tools to detect IPV during pregnancy**	
WHO’s domestic violence questionnaire	12 studies [28–31, 33, 35, 37–39, 42, 45, 50]
Abuse Assessment Screen	2 studies [43, 44]
Conflict Tactics Scale	1 study [36]
Revised Conflict Tactics Scale (CTS2)	1 study [8]
Hurt, Insult, Threaten and Scream	1 study [46]
Stressful Life Events Scale	1 study [47]
Composite Abuse Scale - Short Form (CAS-SF)	1 study [32]
Their own questionnaires	6 studies [27, 34, 40, 41, 48, 49]

### Impact of IPV during pregnancy on maternal health

Only two studies using a prospective cohort design with adjustment of confounders were identified, and both evaluated postpartum depression as the outcome. Higher odds of postpartum depression were significantly associated with physical IPV [aOR = 2.75 (1.19–6.35)] and sexual IPV [aOR = 1.93 (1.01–3.73)] in Vietnam [31], while in Turkey, lower rates of postpartum depression [aOR = 0.056, (0.014–0.236)] were observed in women not exposed compared to those exposed to domestic violence during pregnancy [27].

In a cross-sectional study from Iran, IPV during pregnancy was significantly associated with postpartum depression in bivariate analysis, but this association became insignificant when adjusted for congenital abnormalities in the child and history of postpartum depression [34]. Another two cross-sectional studies reported a significant association between IPV during pregnancy and antenatal depression [35, 38] (Table 5, Additional file 1).

Qualitative studies revealed how physical IPV during pregnancy resulted in a variety of physical injuries to mothers (swollen reddish face and eyes, burns from hot iron and hot water, open wounds, vaginal tears and bleeding as a result of marital rape), miscarriages and unwanted pregnancy [40]. Depression, suicidal ideation and self-harm were described in association with psychological IPV during pregnancy [42]. One study from Uganda reported the negative impact of IPV during pregnancy on women’ on physical health and financial stability for mothers with HIV, as their husbands prohibited them from picking up HIV medications or going to work [49].

### Impact of IPV during pregnancy on child health

The findings of the seven studies which described the impact of IPV during pregnancy are summarized in Table 2. Three studies, which all used a prospective cohort design with adjustment of confounders evaluated child health outcomes such as low birthweight [28, 29], preterm birth [29] and inadequate breast feeding [30]. Once again, IPV was significantly associated with higher odds of these outcomes.

Findings from the cross-sectional studies suggested that IPV was significantly associated with higher odds of low birth weight [36, 37] or intrauterine growth restriction [8]. One case control study revealed sexual violence during pregnancy (but not psychological or physical violence) increased the odds of neonatal mortality [45].

**Table 2:** Studies reporting associations between IPV during pregnancy and child health (with adjustment of confounders)

**Author and year**	**Country**	**Sample size (n) and setting**	**Outcome**	**Association with outcome**
**Prospective cohort studies**				
Dolatian, Mahmoodi [28]	Iran	400	Birthweight	IPV has indirect effect on birthweight in the path analysis of the model: B= − 0.016
Nguyen, Ngo [29]	Vietnam	1,276	Low birthweight and gestational age	• Physical IPV significantly associated with higher odd of preterm birth: aOR = 5.5 (2.1–14.1). • Physical IPV significantly associated with higher odd of low birth weight: aOR = 5.7 (2.2–14.9).
Ribeiro, Batista [30]	Brazil	1,146	Breastfeeding	The higher HR of not being breastfed within the first year of life was significantly associated with: • Violence (by partners/family members) before/during pregnancy increased: HR = 1.39 (1.03–1.88) • Recurrent physical/emotional/sexual violence during pregnancy: HR = 1.46 (1.11–1.92)
**Cross sectional studies**				
Laelago, Belachew [37]	Ethiopia	183 Inpatient	Low birth weight	All IPV: aOR = 14.3 (5.1–40.7)
Lobato, Reichenheim [8]	Brazil	810 Outpatient	Intrauterine growth restriction	Psychological IPV: aOR = 1.15 (1.07–1.23)
Kana, Safiyan [36]	Nigeria	293 Outpatient	Low birth weight	Physical, psychological and sexual IPV were all significantly associated to higher risk.
**Case control study**				
Wondimye, Bezatu [45]	Ethiopia	103 cases and 412 controls Community-based	Neonatal mortality	• Sexual violence during pregnancy increased the risk of outcome: aOR = 3.20 (1.09–9.33). • Psychological and physical violence during pregnancy were not significantly associated.

*aOR* adjusted odd ratio, *HR *hazard ratio. The range of aOR or HR in the parenthesis in the 95% CI of the aOR or HR

### The relationship between social support on IPV during pregnancy

The studies investigating IPV during pregnancy and social support are summarized in Table 3, and most were cross-sectional in nature. One study from Vietnam suggested that a lack of support during pregnancy tripled the odds of experiencing IPV during pregnancy [41]. Social support also seemed to moderate the impact of IPV during pregnancy. For example, it significantly reduced antenatal depression in Malaysia [40] and both antenatal depression and anxiety in Mexico [47].

In a qualitative study conducted on the Chaga and Pare tribes (northern Tanzania) where wives must live with their partners’ families and children are considered to be the property of male partners, IPV victims could receive emotional or financial support from their own families but were usually not welcome back home [44]. They were often asked to stay in their marriages in the children’s interest. In some instances, families acted as mediators between the women and their partners. However, some family members did advise the victims to leave the relationship [51].

**Table 3:** Studies on the impact of social support on IPV during pregnancy (with adjustment of confounders)

Author and year	Country	Sample size (n) and setting	Outcome	Association with risk of outcome
**Quasi-experimental study**				
Bhushan, Krupp [44]	India, community - based	480 Quasi-experimental study	Antenatal anxiety	Home visit or accompaniment to antenatal care by ASHA^a^ significantly associated with lower odds of outcome. • Home visits: aPR = 0.90 (0.76–0.98). • Accompaniment to antenatal care: aPR = 0.86 (0.78–0.95).
**Cross sectional and other studies**				
Manongi, Rogathi [38]	Tanzania, outpatient	1,116 Cross sectional	Antenatal depression	Emotional support from outside family significantly associated with higher odd of outcome: [aOR = 2.25 (1.26, 4.02)] compared with emotional support from inside family.
Woldetensay, Belachew [46]	Ethiopia	4,680 Community – based Baseline data from a prospective, quasi-experimental cohort study	Antenatal depressive symptoms	Poor social support from friends, families and husband significantly associated with higher odds of outcome: aOR = 1.63 (1.31–2.02).
Nguyen, Ngo [41]	Vietnam	1309 Community – based Cross-sectional study nested within a larger prospective cohort study	IPV during pregnancy	Lack of social support significantly associated with higher odds of: • One - time IPV: aOR = 3.1 (2.4–3.9). • Multiple times: aOR = 2.9 (2.2–3.8).
Nasreen, Rahman [40]	Malaysia, outpatient	904 Cross sectional	Antenatal depression and anxiety	Moderate support [aOR = 0.16 (0.03–0.73)] and high support [aOR = 0.13 (0.03–0.59)] significantly associated with lower odds of depression. Anxiety: family support significantly associated with higher odds: aOR = 1.07 (1.03–1.13).
Woldetsadik, Ayele [43]	Ethiopia, community - based	743 Cross sectional	Antenatal common mental disorders	No significant association between social support or husband support and the outcome.
Navarrete, Nieto [47]	Mexico	210 Outpatient	Antenatal depressive and anxiety symptoms	When social support was introduced into the regression model, the impact of IPV during pregnancy was nullified (odd of depression) or reduced (odd of anxiety)^b^.

*aOR* adjusted odd ratio, *aPR* adjusted prevalence ratio, The range of aOR/aPR in the parenthesis in the 95% CI of the aOR/aPR

^a^Accredited Social Health Activists. ^b^This research is a prospective cohort study but only cross-sectional data obtained during pregnancy is used in this review

### Impact of COVID-19 pandemic on IPV during pregnancy

As our library searches captured articles up to April 2021, the retrieved studies included those conducted during the COVID-19 pandemic in its first year. Specifically, four studies from April to November 2020 (see Table 4). Two studies were online or telephone surveys, reflecting the impact of lockdowns or social distancing on feasible research methods. The other two were facility – based.

The prevalence of domestic violence during pregnancy during the pandemic was 35.2% in Iran [39], and 7.1% in Ethiopia [42]. Only one study from Jordan compared the prevalence of IPV during pregnancy before the pandemic with that during the pandemic, finding that the prevalence before the pandemic was actually higher. Specifically, before the pandemic, the prevalence was 65.1%, 30.7%, and 15.3%, for psychological, physical, and sexual violence, respectively, while it was 50.2%, 13%, and 11.2% during the pandemic [33].

During the pandemic, IPV during pregnancy was found to lower the quality of life in Iran [39], and increase the odds of common mental disorders in South Africa [32]. In Ethiopia, IPV during pregnancy was associated with husband’s alcohol or khat consumption during the pandemic [42].

**Table 4:** Studies on the impact of COVID-19 on IPV during pregnancy

Author and year	Country	Sample size (n) and setting	Setting and timing	What the study wants to determine	Association between outcome and social support
Naghizadeh, Mirghafourvand [39]	Iran	250 Cross sectional	Outpatient 5–8/2020	The prevalence of domestic violence and its relationship with the quality of life of pregnant women during the COVID-19 pandemic	• About 1 in 3 women experienced domestic violence. • Violence victims had significant lower quality of life in the mental health compared to unaffected women: β = 9.3 (3.5 to 15.0), P = 0.002)
Teshome, Gudu [42]	Ethiopia	464 Cross sectional	Outpatient 8–11/2020	The incidence and predictors of IPV during pregnancy during the COVID-19 pandemic	• 7.1% women experienced IPV during pregnancy • IPV during pregnancy was more reported among women who husbands consume Khat [aOR = 3.27 (1.45–7.38)] or alcohol [aOR = 1.52 (1.01–2.28)]
Abujilban, Mrayan [33]	Jordan	215 Cross sectional (online survey)	Community 4/2020	The change in the incidence of IPV during pregnancy before and during the COVID-19 pandemic	The pre-pandemic level of IPV during pregnancy was higher than that during the pandemic (Before: 65.1%, 30.7%, and 15.3%, for psychological, physical, and sexual violence, respectively. During: 50.2%, 13%, 11.2%, respectively).
Abrahams, Boisits [32]	South Africa	885 Cross sectional (Telephone interview)	Outpatient 6–7/2020	The relationship between common mental disorders, food insecurity and IPV during pregnancy during the COVID-19 pandemic	Higher odds of common mental disorders were associated with IPV during pregnancy during the pandemic: • Psychological IPV: aOR = 2.50 (1.32–4.72) • Sexual IPV: 2.70 (1.07–6.80)

*aOR* adjusted odd ratio. The range of aOR in the parenthesis in the 95% CI of the aOR

## Discussion

 This scoping review consolidates existing knowledge regarding the impact of IPV during pregnancy on maternal and child health in LMICs, and identifies how social support and the COVID-19 pandemic may affect IPV during pregnancy in LMICs. Our findings confirm the well-established association between IPV during pregnancy and postpartum depression [4] and identifies additional evidence of the association between IPV during pregnancy and low birth weight, preterm birth and less breastfeeding in the year after birth [28, 30, 52]. The previously recognized protective effect of social support for women experiencing IPV during pregnancy was reflected by the findings of quantitative studies, highlighting the potential of social support interventions for improving perinatal mental health outcomes. However, qualitative evidence revealed that in contexts where proprietal attitudes towards women were common, wider social networks may encourage pregnant women to remain in violent relationships [44]. The number of studies exploring IPV during pregnancy during the COVID-19 pandemic is still small, though a negative impact of the pandemic on the quality of life and the mental health of pregnant women was found in the available literature.

### Impact of IPV during pregnancy on maternal health

In their work on the link between IPV during pregnancy and maternal mental health, Halim et al. suggested that prospective cohort designs with adjustment of confounders are optimal for determining causality [5]. In our scoping review, two such studies reported that physical/sexual IPV and domestic violence during pregnancy were significantly associated with a higher risk of postpartum depression [27, 31]. Generally, our data is congruent with the literature before 2016. A prospective cohort study in 2010 from Brazil found a significant association between psychological IPV during pregnancy and postpartum depression [53] while another prospective cohort study conducted in 2015 in Tanzania revealed a similar finding between any IPV during pregnancy and postpartum depression [54]. There is consistent evidence from LMICs supporting the association between IPV during pregnancy and postpartum depression [55, 56]. The two more recent studies in our review only followed up women until eight [19] or twelve [23] weeks after birth, which is similar to existing literature from before 2016. Whilst it is intensive to continue cohort studies further into the postpartum period, there appears to very little research about the length and severity of depressive episodes during the first year postpartum, or if IPV continues during the postpartum period. Antenatal depression occurs concurrently with IPV during pregnancy, and this association has been identified in studies with cross-sectional designs. The finding of a significant association between IPV during pregnancy and antenatal depression in our review is also aligned with previous literature [57, 58].

Previous literature has identified a range of other maternal health issues associated with IPV during pregnancy [3, 5, 59–62]. For *pregnancy outcomes* (abortion, hemorrhage, placenta abruption, preeclampsia, vaginal delivery vs. cesarean), only premature rupture of membranes was found to be significantly associated with physical/psychological IPV in the studies using prospective cohort designs with adjustment of confounders [63, 64].

### Impact of IPV during pregnancy on child health

For *preterm birth*, a study from Vietnam in our review identified a significant association between physical IPV during pregnancy and preterm birth [29]. In the previous literature, psychological IPV during pregnancy was not significantly associated with premature birth in a prospective cohort study from Iran in 2014 [64] or South Africa in HIV – infected mothers in 2017 [65]. Domestic violence of any form during pregnancy was also not associated with this outcome in another prospective cohort study from Brazil in 2008 [66]. These findings, including the association between preterm birth and IPV from the more recent study in Vietnam, can be used to inform interventions to protect maternal and child health in LMICs.

Regarding *low birth weight*, any IPV for the study from Iran [28], and physical IPV for the study from Vietnam [29] were found to be significantly associated. Our findings are consistent prospective cohort studies from Brazil in 2010 (physical IPV during pregnancy, alone or together with psychological IPV) [67] and Iran (physical/sexual IPV) in 2015 [63]. Interestingly, psychological IPV alone during pregnancy was not significantly associated with low birth weight in the previous studies from Iran [64] and South Africa [65]. However, as few prospective cohort studies are available, further research on this association is required.

In terms of *breastfeeding*, high odds of not being breastfed in the first year of life was reported for babies whose mother had been exposed to violence (by partners/family members) before/during pregnancy compared to unexposed mothers [30]. This finding is in keeping with a prospective cohort study from Tanzania which suggests exposure to IPV during either pregnancy or the postpartum period increased the odds of breastfeeding cessation before the child turned 6 months old [68].

We investigated other child outcomes explored in the previous literature [11, 61, 62, 69–71]. However, only one study from China was identified [72] in which the children of the mothers exposed to domestic violence during pregnancy were later found to have poorer *behavioral development* at 10 months of age (weaker rhythmicity, more negative mood, withdrawn behaviour, and poorer development of motor skills). The findings of this scoping review suggest that little is still known about the long-term effects of IPV during pregnancy on child health and development in LMIC settings.

### Impact of social support during pregnancy on IPV during pregnancy

In our review, the association between a lack of social support and IPV during pregnancy is in keeping with the existing literature [13–15]. Social support also seemed to buffer the maternal health impacts of IPV during pregnancy (such as depression and anxiety). However, findings from cross sectional studies should be interpreted with caution due to the inability to conclude on the direction of the association. For example, in our review, the study by Nasreen et al. [40] showed that social support was significantly associated with lower odds of depression, but also with higher odds of anxiety. Further context specific qualitative research is required to understand how different forms of social support can be protective to women in violent relationships during pregnancy in LMIC. It is important to distinguish social support (support from a partner, family or friends [38, 40, 41, 43, 46, 47]) from social support interventions (such as home visits or accompaniment to antenatal care [44]), because the latter are sometimes called “social support” in the literature. For instance, to deliver efficient social support interventions for a pregnant woman experiencing IPV, health professionals should first find out about how much support she has from her partner, family or friends [73]. In India, there are also promising interventions suggesting that social support in the form of home visitors or accompaniment to antenatal care can decrease anxiety [37]. It is acknowledged that globally, the feasibility of such interventions have been seriously disrupted by the COVID-19 pandemic.

### Impact of COVID-19 pandemic on IPV during pregnancy

Evidence from high income countries suggests that the COVID-19 pandemic has worsened the mental health of the general population [74] and also increased the incidence of violence against women [75]. There is currently very little evidence describing how IPV during pregnancy was/is experienced during the pandemic. Surprisingly, in our review, the only study providing a pre and during - pandemic comparison found that the prevalence of IPV during pregnancy throughout the pandemic was lower than that before the pandemic in [33]. Two other studies provided prevalence without such comparison [39, 42]. However, if their figures are compared with the existing data in the literature, the same trend is observed. Specifically, in Iran, the prevalence of domestic violence during pregnancy during the pandemic was 35.2% in our review [39], much lower than the prevalence of 67–70% found in a 2020 study in the same city [76]. In Ethiopia, while the prevalence of IPV during pregnancy was 7.1% in our review, it was as high as 20–35% in other recent studies from this country [35, 77].

Whether this downward trend reflects the true situation during the pandemic or is an artefact of nonrepresentative data (e.g. due to selection bias from online recruitment data collection [33], facility – based sampling [42], urban vs. rural setting [42] or economic and sociocultural differences [39]) is still to be determined. Nevertheless, evidence also suggests that different the pandemic situations in individual countries and the local measures available to combat violence will result in differences in prevalence of violence against women. To date, reports of the rates of domestic violence during the pandemic have been inconsistent, and it is well known that violence against women is underreported in most contexts [17]. According to one multi-country study, domestic violence increased in Austria, Belgium, France, Ireland, Spain and UK, decreased in Italy and Portugal and stayed the same in The Netherlands and Switzerland [78].

The scarcity of research on IPV during pregnancy during the COVID-19 pandemic in LMICs suggests that more research is required to illuminate the needs of pregnant women during this time. Such research is urgently needed due to the serious impact of IPV on pregnant women and their children [32, 39]. Also, whilst social support was found to be protective for women experiencing IPV in pregnancy, globally, lockdown orders and social distancing measures have created changes to maternity care that have resulted in barriers to such support being accessed. In addition, while our review describes the pandemic in 2020, new variants with more serious consequences and/or higher rates of infection [79, 80] mean that women suffering IPV during pregnancy in LMICs will continue to experience the context of the pandemic from 2021 into the future.

## Conclusions

By looking specifically at prospective cohort studies with adjustment of confounders in this review and similar previous reviews, we conclude that high quality literature from LMICs is still limited. The scarcity of research is evident in both the number of studies and types of outcomes evaluated. The following recommendations are thus made as below:

- More longitudinal primary studies with appropriate statistical techniques and longer postpartum timeframes should be conducted to gather more reliable data. Systematic reviews and meta – analysis in this area are unlikely to add new information at this stage.
- Context specific qualitative research is required for nuanced understanding about what types of social support can be protective for pregnant women experiencing IPV.
- Efforts should be made to standardize tools to detect IPV during pregnancy, screening practice and methodology to allow more comparable research.
- The small number of LMICs in our review and others raises the possibility that relevant work might been published in languages other than English. For instance, one review included a few primary studies published in Portuguese [61]. As this possibility is highly true, the need to understand this topic in English literature will require collaboration from authors of LMICs to capture relevant research.
- We believe that different COVID-19 variants are and will be creating diverse impacts on IPV during pregnancy in various stages of the ongoing pandemic and parts of the world. Therefore, scoping reviews are regularly required to inform on the literature available.
- Social support interventions that are feasible during pandemic restrictions in LMIC should be trialed and evaluated, as these are imperative to the wellbeing of pregnant women.

## Supplementary Information

**Additional file 1.**

## Data Availability

Data sharing is not applicable to this article as no datasets were generated or analysed during the current study.

## Abbreviations

IPV: intimate partner violence
LMICs: low- and middle-income countries
GNI: gross national income
