# Correction: Intimate partner violence during pregnancy and maternal and child health outcomes: a scoping review of the literature from low-and-middle income countries from 2016 – 2021

**DOI:** 10.1186/s12884-022-05117-9

**Published:** 2022-10-21

**Authors:** Thao Da Thi Tran, Linda Murray, Thang Van Vo

**Affiliations:** 1 School of Health Sciences, College of Health, Massey University, Wellington, Aotearoa New Zealand; 2 Institute for Community Health Research, University of Medicine and Pharmacy, Hue University, Hue City, Vietnam; 3 Faculty of Public Health, University of Medicine and Pharmacy, Hue University, Hue City, Vietnam

**Correction: BMC Pregnancy Childbirth 22, 315 (2022)**

**https://doi.org/10.1186/s12884-022-04604-3**

Following publication of the original article [1], the authors reported an error in the information in the PRISMA diagram (Fig. 1).

**Fig. 1:**
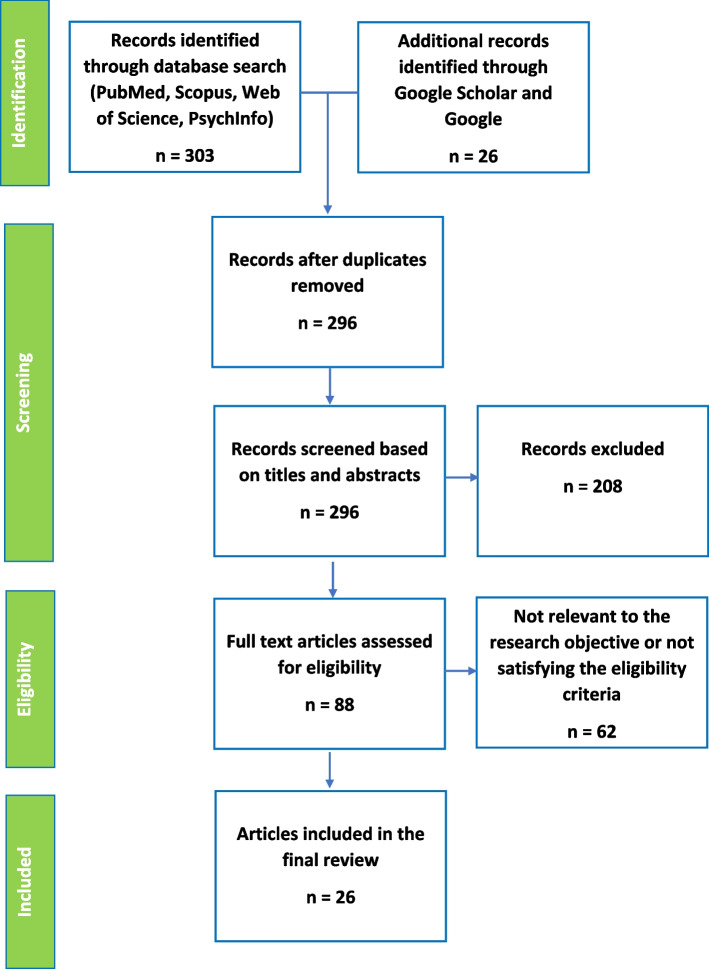
PRISMA flow chart of the study

The original article [1] has been updated.
