# Supplementary material for: Intimate partner violence during pregnancy and maternal and child health outcomes: a scoping review of the literature from low-and-middle income countries from 2016 - 2021
Source: BMC Pregnancy Childbirth. 2022 Apr 13;22:315. doi: 10.1186/s12884-022-04604-3 (PMC9006493; doi:10.1186/s12884-022-04604-3)
Supplement: Supplementary file 1 — Additional file 1. [file 12884_2022_4604_MOESM1_ESM.docx]

## Additional file 1

| **Table 5. Studies on the impact of IPV during pregnancy on maternal health (with adjustment of confounders)** | | | | |
| --- | --- | --- | --- | --- |
| **Author and year** | **Country** | **Sample size (n) and setting** | **Outcome** | **Association with outcome** |
| **Prospective cohort studies** | | | | |
| Tran (2019) [23] | Vietnam | 1,274  Community-based | Postpartum depression | - Physical IPV [aOR= 2.75 (1.19-6.35)] and sexual IPV [aOR= 1.93 (1.01-3.73)] significantly associated with higher risk. - Emotional IPV not significantly associated. |
| Çankaya (2020) [19] | Turkey | 245  Outpatient | Postpartum depression | Odds of outcome was higher in those exposed to domestic violence from husband or another male in the family compared to unexposed women:  aOR = 0.056, (0.014–0.236) |
| **Cross sectional studies** | | | | |
| Manongi (2017) [31] | Tanzania | 1,116  Outpatient | Prenatal depression | - Exposure to any type of IPV: aOR=5.06 (3.25-7.86) - Physical IPV: aOR=4.42 (2.65 - 7.37) - Sexual IPV: aOR= 2.53 (1.60 - 4.00) - Emotional IPV: aOR=2.16 (1.35 - 3.44) |
| Belay (2019) [27] | Ethiopia | 589  Community - based | Prenatal depression | Exposure to any type of IPV: aOR=17.60 (6.18-50.10) |
| Afshari (2020) [26] | Iran | 505  Outpatient | Postpartum depression | Physical IPV: not significant |
| aOR: adjusted odd ratio. The range of aOR in the parenthesis in the 95% CI of the aOR | | | | |

**Supplementary File 1: Search Strategy**

1. **PUBMED**

| **MeSH term and the text words used for the search in Pubmed** | | | |
| --- | --- | --- | --- |
|  | **Measures** | **MeSH terms** | **Text words** |
| P | Pregnant women | Pregnancy  Pregnant women | Antenatal  Pregnant  Reproductive  Maternal  Partum |
|  | Partner supportiveness | Spouses  Male  Interpersonal Relations | Partner support  Husband support  Spouse support  Spousal support  Marital support |
|  | Social support | Social Support | Community support |
|  | Intimate partner violence | Intimate Partner Violence  Domestic violence  Gender-Based Violence  Physical abuse  Exposure to violence  Sex Offenses  Emotional Abuse  Aggression | IPV  Intimate partner abuse  Intimate violence  Partner violence  Intimate terrorism  Partner abuse  Domestic abuse  Wife abuse  Wife beating  Spousal abuse  Spouse violence  Spousal violence  Family violence  Couple violence  Marital violence  Marital abuse  Physical abuse  Sexual violence  Sexual abuse  Emotional violence  Emotional abuse  Psychological violence  Psychological abuse  Abuse during pregnancy |
|  | Living during the Covid-19 pandemic | COVID-19  SARS-CoV-2 | Covid  Corona* |
| O | Mother’s well - being | Mothers  Female  Spouses  Mental health  Mental disorders  Anxiety disorders  Posttraumatic stress disorders  Bipolar disorder  Mania  Social phobia  Depression  Depressive disorders  Postpartum depression  Mood disorders  Psychotic Disorders  Schizophrenia  Neuropsychology  Neurocognitive disorders  Suicide  Chronic fatigue syndrome  Fatigue  Panic disorder  Panic Disorder  Somatoform disorders  Self-control  Substance-Related Disorders  Stress Disorders | Maternal  Female partner  Female spouse  Wife  Anxiety  Social anxiety disorder  Bipolar  Psychosis  Neurocognitive  Suicide risk  Suicidality  Somatic complaints  Substance abuse  Stress |
|  | Birth outcomes  Child health and well-being | Pregnancy complications  Pregnancy outcome  Infant  Infant, Low Birth Weight  Premature birth  Child  Infant behavior  Child Development  Child Rearing  Child Welfare  Neurodevelopmental Disorders  Child Behavior Disorders  Child Development Disorders  Defense Mechanisms  Emotional Regulation  Emotions  Cognition | Toddler  Newborn  Baby  Babies  Preschool  Prenatal complications  Perinatal complications  Birth outcomes  Low Birth Weight  Socio-emotional development  Neurological development  Emotional development  Cognitive development  Childhood trauma |
| Study design | Observational designs (cohort, case control, cross – sectional, case series) | Epidemiologic studies  Cohort Studies  Case-Control Studies  Cross-Sectional Studies | Cohort  Case-control  Cross sectional  Cross-sectional  Cases  Survey  Prospective  Retrospective  Epidemiologic* |

Search strings

| 1 | Pregnancy[MeSH] OR Pregnant women[MeSH] OR Pregnancy[tiab] OR Pregnant women[tiab] OR Antenatal[tiab] OR Pregnant[tiab] OR Reproductive[tiab] OR Maternal[tiab] OR Partum[tiab] |
| --- | --- |
| 2 | ((Spouses[MeSH] AND Male[MeSH]) OR (Interpersonal Relations[MeSH])) OR (Spouses[tiab] OR Male[tiab] OR Interpersonal Relations[tiab] OR Partner support[tiab] OR Husband support[tiab] OR Spouse support[tiab] OR Spousal support[tiab] OR Marital support[tiab]) |
| 3 | Social Support[MeSH] OR Social Support[tiab] OR Community support[tiab] |
| 4 | Intimate Partner Violence[MeSH] OR Domestic violence[MeSH] OR Gender-Based Violence[MeSH] OR Physical abuse[MeSH] OR Exposure to violence[MeSH] OR Sex Offenses[MeSH] OR Emotional Abuse[MeSH] OR Aggression[MeSH] OR Intimate Partner Violence[tiab] OR Domestic violence[tiab] OR Gender-Based Violence[tiab] OR Physical abuse[tiab] OR Exposure to violence[tiab] OR Sex Offenses[tiab] OR Emotional Abuse[tiab] OR Aggressi*[tiab] OR IPV[tiab] OR Intimate partner abuse[tiab] OR Intimate violence[tiab] OR Partner violence[tiab] OR Intimate terrorism[tiab] OR Partner abuse[tiab] OR Domestic abuse[tiab] OR Wife abuse[tiab] OR Wife beating[tiab] OR Spousal abuse[tiab] OR Spouse violence[tiab] OR Spousal violence [tiab] OR Family violence[tiab] OR Couple violence [tiab] OR Marital violence[tiab] OR Marital abuse[tiab] OR Physical abuse[tiab] OR Sexual violence[tiab] OR Sexual abuse[tiab] OR Emotional violence[tiab] OR Emotional abuse[tiab] OR Psychological violence[tiab] OR Psychological abuse[tiab] OR Abuse during pregnancy[tiab] |
| 5 | COVID-19[MeSH] OR SARS-CoV-2[MeSh] OR COVID-19[tiab] OR SARS-CoV-2[tiab] OR Covid[tiab] OR corona*[tiab] |
| 6 | (Mothers[MeSH] OR female[MeSH] OR spouses [MeSH] OR mother[tiab] OR female[tiab] OR spouse*[tiab] OR maternal[tiab] OR female part[tiab] OR female spouse[tiab] OR wife[tiab]) OR (Mental health[MeSH] OR Mental disorders[MeSH] OR Anxiety disorders[MeSH] OR Posttraumatic stress disorders[MeSH] OR Bipolar disorder[MeSH] OR Mania[MeSH] OR Social phobia[MeSH] OR Depression[MeSH] OR Depressive disorders[MeSH] OR Postpartum depression[MeSH] OR Mood disorders[MeSH] OR Psychotic Disorders[MeSH] OR Schizophrenia[MeSH] OR Neuropsychology[MeSH] OR Neurocognitive disorders[MeSH] OR Suicide[MeSH] OR Chronic fatigue syndrome[MeSH] OR Fatigue[MeSH] OR Panic disorder[MeSH] OR Panic Disorder[MeSH] OR Somatoform disorders[MeSH] OR Self-control[MeSH] OR Substance-Related Disorders[MeSH] OR Stress Disorders[MeSH] OR Mental health[tiab] OR Mental disorders[tiab] OR Anxiety disorders[tiab] OR Posttraumatic stress disorders[tiab] OR Bipolar disorder[tiab] OR Mania[tiab] OR Social phobia[tiab] OR Depression[tiab] OR Depressive disorders[tiab] OR Postpartum depression[tiab] OR Mood disorders[tiab] OR Psychotic Disorders[tiab] OR Schizophrenia[tiab] OR Neuropsychology[tiab] OR Neurocognitive disorders[tiab] OR Suicide[tiab] OR Chronic fatigue syndrome[tiab] OR Fatigue[tiab] OR Panic disorder[tiab] OR Panic Disorder[tiab] OR Somatoform disorders[tiab] OR Self-control[tiab] OR Substance-Related Disorders[tiab] OR Stress Disorders[tiab] OR Anxiety[tiab] OR Social anxiety disorder[tiab] OR Bipolar[tiab] OR Psychosis[tiab] OR Neurocognitive[tiab] OR Suicide risk[tiab] OR Suicidality[tiab] OR Somatic complaints[tiab] OR Substance abuse[tiab] OR Stress[tiab]) |
| 7 | Pregnancy complications[MeSH] OR Pregnancy outcome[MeSH] OR Infant[MeSH] OR Low Birth Weight[MeSH] OR Premature birth[MeSH] OR Child[MeSH] OR Infant behavior[MeSH] OR Child Development[MeSH] OR Child Rearing[MeSH] OR Child Welfare[MeSH] OR Neurodevelopmental Disorders[MeSH] OR Child Behavior Disorders[MeSH] OR Child Development Disorders[MeSH] OR Defense Mechanisms[MeSH] OR Emotional Regulation[MeSH] OR Emotions[MeSH] OR Cognition[MeSH] OR Pregnancy complications[tiab] OR Pregnancy outcome[tiab] OR Infant[tiab] OR Child[tiab] OR Preschool[tiab] OR baby[tiab] OR babies[tiab] OR Infant behavio*[tiab] OR Child Development[tiab] OR Child Rearing[tiab] OR Child Welfare[tiab] OR Neurodevelopmental Disorders[tiab] OR Child Behavior Disorders[tiab] OR Child Development Disorders[tiab] OR Defense Mechanism*[tiab] OR Emotional Regulation[tiab] OR Emotion*[tiab] OR Cogniti*[tiab] OR Toddler[tiab] OR Newborn[tiab] OR Prenatal complication*[tiab] OR Perinatal complication*[tiab] OR Birth outcome*[tiab] OR Premature birth[tiab] OR Low Birth Weight[tiab] OR Socio-emotional development[tiab] OR Neurological development[tiab] OR Emotional development[tiab] OR Cognitive development[tiab] OR Childhood trauma[tiab] |
| 8 | Epidemiologic studies[MeSH] OR Cohort Studies[MeSH] OR Case-Control Studies[MeSH] OR Cross-Sectional Studies[MeSH] OR Epidemiologic studies[tiab] OR Cohort Study[tiab] OR Case-Control Study[tiab] OR Cross-Sectional Study[tiab] OR Cohort[tiab] OR Case-control[tiab] OR Case control[tiab] OR Cross sectional[tiab] OR Cross-sectional[tiab] OR Cases[tiab] OR Survey[tiab] OR Prospective[tiab] OR Retrospective[tiab] OR Epidemiologic*[tiab] |
| 9 | #2 OR #3 OR #5 |
| 10 | #1 AND #4 AND #6 AND #7 AND #8 AND #9 |
| 11 | Limit the results to 1/2015 – present |

1. **SCOPUS**

| 1 | TITLE-ABS-KEY(pregnan* OR "Pregnant wom?n" OR antenatal OR reproducti* OR maternal OR partum) |
| --- | --- |
| 2 | TITLE-ABS-KEY((Partner* OR Husband OR Spous* OR Marital) AND support*) |
| 3 | TITLE-ABS-KEY("Social Support" OR "Community support") |
| 4 | TITLE-ABS-KEY(“Intimate Partner Violence” OR “Domestic violence” OR “Gender-Based Violence” OR “Physical abuse” OR “Exposure to violence” OR “Sex Offenses” OR “Emotional Abuse” OR Aggression OR IPV OR “Intimate partner abuse” OR “Intimate violence” OR “Partner violence” OR “Intimate terrorism” OR “Partner abuse” OR “Domestic abuse” OR “Wife abuse” OR “Wife beating” OR “Spousal abuse” OR “Spouse violence” OR “Spousal violence” OR “Family violence” OR “Couple violence” OR “Marital violence” OR “Marital abuse” OR “Physical abuse” OR “Sexual violence” OR “Sexual abuse” OR “Emotional violence” OR “Emotional abuse” OR “Psychological violence” OR “Psychological abuse” OR “Abuse during pregnancy”) |
| 5 | TITLE-ABS-KEY(COVID-19 OR SARS-CoV-2 OR Covid OR Corona*) |
| 6 | TITLE-ABS-KEY((mother OR female OR spouse* OR maternal OR “female spouse” OR “female partner” OR wife) AND ("Mental health" OR "Mental disorder*" OR "Anxiety disorder*" OR "Posttraumatic stress disorder*" OR PTSD OR "Bipolar disorder" OR Mania OR "Social phobia" OR Depress* OR "Depressive disorder*" OR "Postpartum depression" OR "Mood disorder*" OR "Psychotic disorder*" OR Schizophrenia OR Neuropsychology OR "Neurocognitive disorder*" OR Suicid* OR "Chronic fatigue syndrome" OR Fatigue* OR "Panic disorder*" OR "Somatoform disorder*" OR Self-control OR "Substance-Related Disorder*" OR "Stress Disorder*" OR Anxiety OR "Social anxiety disorder*" OR Bipolar OR Psychosis OR Neurocognitive OR "Suicide risk*" OR "Somatic complaint*" OR "Substance abuse" OR Stress)) |
| 7 | TITLE-ABS-KEY((Infant* OR Toddler* OR Newborn* OR Preschool OR Child* OR baby OR babies) AND ("Pregnancy complication*" OR "Pregnancy outcome*" OR "Infant behavio?r" OR "Child Development" OR "Child Rearing" OR "Child Welfare" OR "Neurodevelopmental Disorder*" OR "Child Behavio?r Disorder*" OR "Child Development Disorder*" OR "Defense Mechanism*" OR "Emotional Regulation*" OR Emotion* OR Cogniti* OR "Prenatal complication*" OR "Perinatal complication*" OR "Birth outcome*" OR "Premature birth*" OR "Low Birth Weight" OR "Socio-emotional development" OR "Neurological development" OR "Emotional development" OR "Cognitive development" OR "Childhood trauma")) |
| 8 | TITLE-ABS-KEY(“Epidemiologic stud*” OR “Cohort Stud*” OR “Case-Control Stud*” OR “Cross-Sectional Stud*” OR Cohort OR “Case-control” OR “Case control” OR “Cross sectional” OR “Cross-sectional” OR Cases OR Survey* OR Prospective OR Retrospective OR Epidemiologic*) |
| 9 | #2 OR #3 OR #5 |
| 10 | #1 AND #4 AND #6 AND #7 AND #8 AND #9 |
| 11 | Limit the results to 1/2015 – present |

1. **WEB OF SCIENCE**

| 1 | TI=(pregnan* OR "Pregnant wom?n" OR antenatal OR reproducti* OR maternal OR partum) AND AB=(pregnan* OR "Pregnant wom?n" OR antenatal  OR reproducti* OR maternal OR partum) |
| --- | --- |
| 2 | TI=((Partner* OR Husband OR Spous* OR Marital) AND support*) OR AB=((Partner* OR Husband OR Spous* OR Marital) AND support*) |
| 3 | TI=("Social Support" OR "Community support") OR AB=("Social Support" OR "Community support") |
| 4 | TI=(“Intimate Partner Violence” OR “Domestic violence” OR “Gender-Based Violence” OR “Physical abuse” OR “Exposure to violence” OR “Sex Offenses” OR “Emotional Abuse” OR Aggression OR IPV OR “Intimate partner abuse” OR “Intimate violence” OR “Partner violence” OR “Intimate terrorism” OR “Partner abuse” OR “Domestic abuse” OR “Wife abuse” OR “Wife beating” OR “Spousal abuse” OR “Spouse violence” OR “Spousal violence” OR “Family violence” OR “Couple violence” OR “Marital violence” OR “Marital abuse” OR “Physical abuse” OR “Sexual violence” OR “Sexual abuse” OR “Emotional violence” OR “Emotional abuse” OR “Psychological violence” OR “Psychological abuse” OR “Abuse during pregnancy”) OR AB=(“Intimate Partner Violence” OR “Domestic violence” OR “Gender-Based Violence” OR “Physical abuse” OR “Exposure to violence” OR “Sex Offenses” OR “Emotional Abuse” OR Aggression OR IPV OR “Intimate partner abuse” OR “Intimate violence” OR “Partner violence” OR “Intimate terrorism” OR “Partner abuse” OR “Domestic abuse” OR “Wife abuse” OR “Wife beating” OR “Spousal abuse” OR “Spouse violence” OR “Spousal violence” OR “Family violence” OR “Couple violence” OR “Marital violence” OR “Marital abuse” OR “Physical abuse” OR “Sexual violence” OR “Sexual abuse” OR “Emotional violence” OR “Emotional abuse” OR “Psychological violence” OR “Psychological abuse” OR “Abuse during pregnancy”) |
| 5 | TI=(COVID-19 OR SARS-CoV-2 OR Covid OR Corona*) OR AB=(COVID-19 OR SARS-CoV-2 OR Covid OR Corona*) |
| 6 | TI=((mother OR female OR spouse* OR maternal OR “female spouse” OR “female partner” OR wife) AND ("Mental health" OR "Mental disorder*" OR "Anxiety disorder*" OR "Posttraumatic stress disorder*" OR PTSD OR "Bipolar disorder" OR Mania OR "Social phobia" OR Depress* OR "Depressive disorder*" OR "Postpartum depression" OR "Mood disorder*" OR "Psychotic disorder*" OR Schizophrenia OR Neuropsychology OR "Neurocognitive disorder*" OR Suicid* OR "Chronic fatigue syndrome" OR Fatigue* OR "Panic disorder*" OR "Somatoform disorder*" OR Self-control OR "Substance-Related Disorder*" OR "Stress Disorder*" OR Anxiety OR "Social anxiety disorder*" OR Bipolar OR Psychosis OR Neurocognitive OR "Suicide risk*" OR "Somatic complaint*" OR "Substance abuse" OR Stress)) OR AB=((mother OR female OR spouse* OR maternal OR “female spouse” OR “female partner” OR wife) AND ("Mental health" OR "Mental disorder*" OR "Anxiety disorder*" OR "Posttraumatic stress disorder*" OR PTSD OR "Bipolar disorder" OR Mania OR "Social phobia" OR Depress* OR "Depressive disorder*" OR "Postpartum depression" OR "Mood disorder*" OR "Psychotic disorder*" OR Schizophrenia OR Neuropsychology OR "Neurocognitive disorder*" OR Suicid* OR "Chronic fatigue syndrome" OR Fatigue* OR "Panic disorder*" OR "Somatoform disorder*" OR Self-control OR "Substance-Related Disorder*" OR "Stress Disorder*" OR Anxiety OR "Social anxiety disorder*" OR Bipolar OR Psychosis OR Neurocognitive OR "Suicide risk*" OR "Somatic complaint*" OR "Substance abuse" OR Stress)) |
| 7 | TI=((Infant* OR Toddler* OR Newborn* OR Preschool OR Child* OR baby OR babies) AND ("Pregnancy complication*" OR "Pregnancy outcome*" OR "Infant behavio?r" OR "Child Development" OR "Child Rearing" OR "Child Welfare" OR "Neurodevelopmental Disorder*" OR "Child Behavio?r Disorder*" OR "Child Development Disorder*" OR "Defense Mechanism*" OR "Emotional Regulation*" OR Emotion* OR Cogniti* OR "Prenatal complication*" OR "Perinatal complication*" OR "Birth outcome*" OR "Premature birth*" OR "Low Birth Weight" OR "Socio-emotional development" OR "Neurological development" OR "Emotional development" OR "Cognitive development" OR "Childhood trauma")) OR AB=((Infant OR Toddler OR Newborn OR Preschool OR Child* OR baby OR babies) AND ("Pregnancy complication*" OR "Pregnancy outcome*" OR "Infant behavio?r" OR "Child Development" OR "Child Rearing" OR "Child Welfare" OR "Neurodevelopmental Disorder*" OR "Child Behavio?r Disorder*" OR "Child Development Disorder*" OR "Defense Mechanism*" OR "Emotional Regulation*" OR Emotion* OR Cogniti* OR "Prenatal complication*" OR "Perinatal complication*" OR "Birth outcome*" OR "Premature birth*" OR "Low Birth Weight" OR "Socio-emotional development" OR "Neurological development" OR "Emotional development" OR "Cognitive development" OR "Childhood trauma")) |
| 8 | TI=(“Epidemiologic stud*” OR “Cohort Stud*” OR “Case-Control Stud*” OR “Cross-Sectional Stud*” OR Cohort OR “Case-control” OR “Case control” OR “Cross sectional” OR “Cross-sectional” OR Cases OR Survey* OR Prospective OR Retrospective OR Epidemiologic*) OR AB=(“Epidemiologic stud*” OR “Cohort Stud*” OR “Case-Control Stud*” OR “Cross-Sectional Stud*” OR Cohort OR “Case-control” OR “Case control” OR “Cross sectional” OR “Cross-sectional” OR Cases OR Survey* OR Prospective OR Retrospective OR Epidemiologic*) |
| 9 | #2 OR #3 OR #5 |
| 10 | #1 AND #4 AND #6 AND #7 AND #8 AND #9 |

Each search is restricted to articles (document type) and from 1/2015 to present (timespan).

1. **PsycINFO (via EBSCO as per electronic Massey Library)**

| 1 | TI(pregnan* OR "Pregnant wom?n" OR antenatal OR reproducti* OR maternal OR partum) AND AB(pregnan* OR "Pregnant wom?n" OR antenatal  OR reproducti* OR maternal OR partum) |
| --- | --- |
| 2 | TI((Partner* OR Husband OR Spous* OR Marital) AND support*) OR AB((Partner* OR Husband OR Spous* OR Marital) AND support*) |
| 3 | TI("Social Support" OR "Community support") OR AB("Social Support" OR "Community support") |
| 4 | TI(“Intimate Partner Violence” OR “Domestic violence” OR “Gender-Based Violence” OR “Physical abuse” OR “Exposure to violence” OR “Sex Offenses” OR “Emotional Abuse” OR Aggression OR IPV OR “Intimate partner abuse” OR “Intimate violence” OR “Partner violence” OR “Intimate terrorism” OR “Partner abuse” OR “Domestic abuse” OR “Wife abuse” OR “Wife beating” OR “Spousal abuse” OR “Spouse violence” OR “Spousal violence” OR “Family violence” OR “Couple violence” OR “Marital violence” OR “Marital abuse” OR “Physical abuse” OR “Sexual violence” OR “Sexual abuse” OR “Emotional violence” OR “Emotional abuse” OR “Psychological violence” OR “Psychological abuse” OR “Abuse during pregnancy”) OR AB(“Intimate Partner Violence” OR “Domestic violence” OR “Gender-Based Violence” OR “Physical abuse” OR “Exposure to violence” OR “Sex Offenses” OR “Emotional Abuse” OR Aggression OR IPV OR “Intimate partner abuse” OR “Intimate violence” OR “Partner violence” OR “Intimate terrorism” OR “Partner abuse” OR “Domestic abuse” OR “Wife abuse” OR “Wife beating” OR “Spousal abuse” OR “Spouse violence” OR “Spousal violence” OR “Family violence” OR “Couple violence” OR “Marital violence” OR “Marital abuse” OR “Physical abuse” OR “Sexual violence” OR “Sexual abuse” OR “Emotional violence” OR “Emotional abuse” OR “Psychological violence” OR “Psychological abuse” OR “Abuse during pregnancy”) |
| 5 | TI(COVID-19 OR SARS-CoV-2 OR Covid OR Corona*) OR AB(COVID-19 OR SARS-CoV-2 OR Covid OR Corona*) |
| 6 | TI=((mother OR female OR spouse* OR maternal OR “female spouse” OR “female partner” OR wife) AND ("Mental health" OR "Mental disorder*" OR "Anxiety disorder*" OR "Posttraumatic stress disorder*" OR PTSD OR "Bipolar disorder" OR Mania OR "Social phobia" OR Depress* OR "Depressive disorder*" OR "Postpartum depression" OR "Mood disorder*" OR "Psychotic disorder*" OR Schizophrenia OR Neuropsychology OR "Neurocognitive disorder*" OR Suicid* OR "Chronic fatigue syndrome" OR Fatigue* OR "Panic disorder*" OR "Somatoform disorder*" OR Self-control OR "Substance-Related Disorder*" OR "Stress Disorder*" OR Anxiety OR "Social anxiety disorder*" OR Bipolar OR Psychosis OR Neurocognitive OR "Suicide risk*" OR "Somatic complaint*" OR "Substance abuse" OR Stress)) OR AB=((mother OR female OR spouse* OR maternal OR “female spouse” OR “female partner” OR wife) AND ("Mental health" OR "Mental disorder*" OR "Anxiety disorder*" OR "Posttraumatic stress disorder*" OR PTSD OR "Bipolar disorder" OR Mania OR "Social phobia" OR Depress* OR "Depressive disorder*" OR "Postpartum depression" OR "Mood disorder*" OR "Psychotic disorder*" OR Schizophrenia OR Neuropsychology OR "Neurocognitive disorder*" OR Suicid* OR "Chronic fatigue syndrome" OR Fatigue* OR "Panic disorder*" OR "Somatoform disorder*" OR Self-control OR "Substance-Related Disorder*" OR "Stress Disorder*" OR Anxiety OR "Social anxiety disorder*" OR Bipolar OR Psychosis OR Neurocognitive OR "Suicide risk*" OR "Somatic complaint*" OR "Substance abuse" OR Stress)) |
| 7 | TI((Infant* OR Toddler* OR Newborn* OR Preschool OR Child* OR baby OR babies) AND ("Pregnancy complication*" OR "Pregnancy outcome*" OR "Infant behavio?r" OR "Child Development" OR "Child Rearing" OR "Child Welfare" OR "Neurodevelopmental Disorder*" OR "Child Behavio?r Disorder*" OR "Child Development Disorder*" OR "Defense Mechanism*" OR "Emotional Regulation*" OR Emotion* OR Cogniti* OR "Prenatal complication*" OR "Perinatal complication*" OR "Birth outcome*" OR "Premature birth*" OR "Low Birth Weight" OR "Socio-emotional development" OR "Neurological development" OR "Emotional development" OR "Cognitive development" OR "Childhood trauma")) OR AB((Infant OR Toddler OR Newborn OR Preschool OR Child* OR baby OR babies) AND ("Pregnancy complication*" OR "Pregnancy outcome*" OR "Infant behavio?r" OR "Child Development" OR "Child Rearing" OR "Child Welfare" OR "Neurodevelopmental Disorder*" OR "Child Behavio?r Disorder*" OR "Child Development Disorder*" OR "Defense Mechanism*" OR "Emotional Regulation*" OR Emotion* OR Cogniti* OR "Prenatal complication*" OR "Perinatal complication*" OR "Birth outcome*" OR "Premature birth*" OR "Low Birth Weight" OR "Socio-emotional development" OR "Neurological development" OR "Emotional development" OR "Cognitive development" OR "Childhood trauma")) |
| 8 | TI(“Epidemiologic stud*” OR “Cohort Stud*” OR “Case-Control Stud*” OR “Cross-Sectional Stud*” OR Cohort OR “Case-control” OR “Case control” OR “Cross sectional” OR “Cross-sectional” OR Cases OR Survey* OR Prospective OR Retrospective OR Epidemiologic*) OR AB(“Epidemiologic stud*” OR “Cohort Stud*” OR “Case-Control Stud*” OR “Cross-Sectional Stud*” OR Cohort OR “Case-control” OR “Case control” OR “Cross sectional” OR “Cross-sectional” OR Cases OR Survey* OR Prospective OR Retrospective OR Epidemiologic*) |
| 9 | #2 OR #3 OR #5 |
| 10 | #1 AND #4 AND #6 AND #7 AND #8 AND #9 |

Each search is restricted to articles from 1/2015 to present

1. **GOOGLE AND GOOGLE SCHOLAR**

The key words used are: pregnancy, pregnant women, intimate partner violence, mother, child, covid.

**Supplementary File 2**

**SCREENING CRITERIA**

After literature search, 329 results were loaded into the online platform Picoportal (picoportal.net).

Picoportal platform provides the PICOS system for screening full texts. We defined PICOS for our scoping review as in the table below.

|  | **Inclusion criteria** | **Exclusion criteria** |
| --- | --- | --- |
| **Population** | Pregnant women OR mothers in low- and middle-income countries | No exposure to prenatal intimate partner violence  The participating women have a known diagnosis/treatment of mental health issues, or overt psychosis or concurrent severe physical health problems.  The participating women have covid infection |
| **Intervention** | Exposure intimate partner violence during pregnancy OR (partner support OR social/community support during pregnancy OR life during covid-19) | Nil |
| **Comparison** | Nil | Nil |
| **Outcomes** | Maternal health/well - being OR child health/development/well-being (up to 18 months) |  |
| **Study type measures** | Observational (including but not limiting to cross-sectional, prospective cohort, case control, case series), qualitative studies, mixed methods studies | Controlled trials, experiment studies |

The LMICs as defined by the World Bank [1]:

- Low income countries are those with a gross national income (GNI) per capita of $1,035 or less.
- Middle income countries have GNI per capita between $1,036 and $12,535.

**REFERENCES**

1. World Bank. World Bank Country and Lending Groups 2021 [Available from: <https://datahelpdesk.worldbank.org/knowledgebase/articles/906519-world-bank-country-and-lending-groups>.

**Supplementary File 3**

**LIST OF DATA ITEMS**

1. Full title of the article

2. Last name of the first author

3. Publication year

4. Country of study

5. Objective of the study

6. Study design

7. Sources of sample

- Community/population
- Outpatients
- Inpatients
- Other

8. Type of participants' abode

9. Inclusion criteria

10. Exclusion criteria

11. Participants

12. Types of mother's well - being issues explored in the study

13. Tool used to assess mother's well - being

- Edinburgh Postnatal Depression Scale (EPDS) for depression
- Patient Health Questionnaire (PHQ-9) for depression
- Hospital Anxiety and Depression Scale (HADS) for depression
- Depression, Anxiety, and Stress Scale (DASS 21) for anxiety
- Edinburgh Postnatal Depression Scale (EPDS) known as EPDS-3A for anxiety
- Perceived Stress Scale for stress
- WHO Disability Assessment Schedule (WHODAS 2.0)
- Other:

14. Timing of IPV

- In the last 6 months of the current pregnancy
- In the last 12 months of the current pregnancy
- Before and during this pregnancy (unclear duration)
- During the current pregnancy
- Other

15. Perpetrators of IPV

- Husband or partner
- Other
- 16. Types of IPV explored in the study
- Physical
- Psychological (emotional)
- Sexual
- Other

17. Tool used to assess IPV

18. The study explored husband/partner's support

- Yes
- No

19. The study explored social/community support

- Yes
- No

20. If the study explored social/community support, what was the tool used to assess this support?

- Multidimensional Scale of Perceived Social Support (MSPSS)
- Maternity Social Support Scale (MSSS)
- Oslo Social Support Scale
- Medical Outcomes Study (MOS)
- Social Support Survey
- Functional Social Support Questionnaire (FSSQ)
- Family Needs Screener
- Interpersonal Support Evaluation List (ISEL-12)
- Norbeck Social Support Questionnaire
- Social/community not explored in this study
- Other

21. The study explored life during Covid-19 pandemic

- Yes
- No

22. Type of pregnancy outcomes/child outcomes explored in the study

23. Tool used to assess pregnancy outcomes/child outcomes

24. Ethics approval

25. Sample size

26. Age of pregnancy (if participants are pregnant women)

OR age of children (if participants are mothers)

27. Age range of participants

28. Prevalence or incidence of mother's well - being issues

29. Prevalence or incidence of IPV during pregnancy.

- Physical
- Psychological (emotional)
- Sexual
- Overall
- Other

30. Predictors of IPV

31. Prevalence or incidence of husband/partner's support

32. Prevalence or incidence of social/community support

33. Prevalence or incidence of the variables on life aspects during Covid-19 pandemic

34. Association between IPV and mother's well - being

- If there is multivariate analysis, write result:

35. Association between husband/partner's support and mother's well - being

- If there is multivariate analysis, write result:

36. Association between social/community support and mother's well - being

- If there is multivariate analysis, write result:

37. Association between life during Covid-19 pandemic and mother's well – being

- If there is multivariate analysis, write result:

38. Is there any other factors significantly associated with mother's well - being?

- If there is multivariate analysis, write result:

39. Prevalence or incidence of pregnancy outcomes/child outcomes

40. Association between IPV and pregnancy outcomes/child outcomes

- If there is multivariate analysis, write result:

41. Is there any other factors significantly associated with pregnancy outcomes/child outcomes?

- If there is multivariate analysis, write result:

**Supplementary File 4**

**PREVALENCE OF IPV DURING PREGNANCY**

The prevalence of IPV during pregnancy was explored in different settings such as national health surveys, community – based, and facility - based. The only national health survey evaluating IPV during pregnancy was conducted in Turkey [1]. Interestingly, the prevalence of psychological IPV was very high and stable over the periods considered: 92.47% in 2008 and 94.6% in 2014. The same trend was also seen for sexual IPV: 49.55% in 2008 and 49.3% in 2014. The community – based studies in two Eastern Africa countries, Rwanda and Ethiopia reported that the prevalence of three types of IPV were about 10% [2, 3]. Conversely, a study in Vietnam revealed a much higher prevalence of psychological IPV, at 32.2%, and much lower prevalence of physical IPV, at 3.5% [4].

With clinics and hospitals being the most common setting for studies, facility – based prevalence was highly variable, within and between countries. Very high figures were observed in one study in Nigeria: any IPV at 67%, physical IPV 34.1%, psychological IPV at 50.2% and sexual IPV at 30.7% [5]. Except the low prevalence of IPV in a study performed during the COVID-19 pandemic [6], most figures were in the 20 – 50% range.

| **Table 1. Prevalence of IPV during pregnancy** | | | | | | | |
| --- | --- | --- | --- | --- | --- | --- | --- |
| **Author and year** | **Country** | **Study design** | **Sample (n)** | **Any IPV (%)** | **Physical IPV (%)** | **Psychological/**  **emotional IPV (%)** | **Sexual IPV (%)** |
| **National Health Survey** | | | | | | | |
| Yüksel-Kaptanoğlu (2019) [1] | Turkey | National Research on Domestic Violence  Against Women in Turkey (2008 and 2014) | 12,795 | - | - | 92.47 (in 2008), 94.6 (in 2014) | 49.55 (in 2008), 49.3 (in 2014) |
| **Community – based** | | | | | | | |
| Rurangirwa (2017) [3] | Rwanda | Cross sectional | 921 | - | 10.2 | 17 | 9.7 |
| Belay (2019) [2] | Ethiopia | Cross sectional | 589 | 21.2 | 9.2 | 14.6 | 9.5 |
| Nguyen (2018) [4] | Vietnam | Cross sectional | 1,309 | 35.2 | 3.5 | 32.2 | 9.9 |
| **Outpatients** | | | | | | | |
| Manongi (2017) [7] | Tanzania | Cross sectional | 1,116 | 38.8 | 10 | 30.7 | 19 |
| Schneider (2018) [8] | South Africa | Baseline data of RCT | 425 | 13.9 | 12.2 | - | 4.2 |
| Gashaw (2018) [9] | Ethiopia | Cross sectional* | 720 | 35.6 | 31.6 | - | - |
| Tesfaye (2021) [10] | Ethiopia | Cross sectional | 314 | - | 19.4 | 16.9 | 5.1 |
| Teshome (2021) [6] | Ethiopia | Cross sectional | 464 | 7.1 | 2.1 | 5.1 | 3.4 |
| Ezeudu (2019) [11] | Nigeria | Mixed method | 702 | 37.2 | 15.8 | 24.5 | - |
| Kana (2020) [5] | Nigeria | Cross sectional | 293 | 67 | 34.1 | 51.2 | 30.7 |
| Nasreen (2018) [12] | Malaysia | Cross sectional | 450 (East Coast), 454 (West Coast) | - | 1.3 (East Coast), 1.5 (West Coast) | - | - |
| Shrestha (2016) [13] | Nepal | Cross sectional | 404 | 27.2 | 3.2 | 16.6 |  |
| Khatoon (2021) [14] | India | Prospective cohort** | 270 | 22.2 | 20 | 50 | 25 |
| Naghizadeh (2021) [15] | Iran | Cross sectional | 250 | 35.2 | 4.8 | 32.8 | 12.4 |
| McKelvie (2020) [16] | Vanuatu | Cross sectional | 192 | 42.2 | 21.93 | 33.69 | 12.43 |
| Lobato (2018) [17] | Brazil | Cross sectional | 810 | - | 37.9 | 82.1 | - |
| Ribeiro (2020) [18] | Brazil | Prospective cohort | 1,146 | - | 12.1 | 47.5 | 2.8 |
| Navarrete (2021) [19] | Mexico | Prospective cohort | 210 | 10.7 | - | - | - |
| **Inpatients** | | | | | | | |
| Laelago (2017) [20] | Ethiopia | Cross sectional | 183 | 23 | 15 | 20 | 12 |
| le Roux (2019) [21] | South Africa | Prospective cohort | 458 | 22 | - | - | - |
| Luhumyo (2020) [22] | Kenya | Cross sectional | 369 | 34.1 | 22.8*** | 27.4 | - |
| Jain (2017) [23] | India | Prospective observational | 400 | 12.3 | 10 | 10.7 | 1.8 |
| Kashanian (2021) [24] | Iran | Cross sectional | 200 | - | 15 | 38 | - |
| Afkhamzadeh (2021) [25] | Iran | Prospective cohort | 1,080 | - | 18.92 | 37.36 | 23.60 |
| *Perpetrators could be partners, stranger, In-laws; **Perpetrators could be partners or family members; ***Physical/sexual IPV | | | | | | | |

**REFERENCES**

1. Yüksel-Kaptanoğlu İ, Adalı T. Intimate Partner Violence During Pregnancy in Turkey: Determinants From Nationwide Surveys. Journal of Interpersonal Violence. 2019:0886260519837652.

2. Belay S, Astatkie A, Emmelin M, Hinderaker SG. Intimate partner violence and maternal depression during pregnancy: A community-based cross-sectional study in Ethiopia. PloS one. 2019;14(7):e0220003-e.

3. Rurangirwa AA, Mogren I, Ntaganira J, Krantz G. Intimate partner violence among pregnant women in Rwanda, its associated risk factors and relationship to ANC services attendance: a population-based study. BMJ Open. 2017;7(2):e013155.

4. Nguyen TH, Ngo TV, Nguyen VD, Nguyen HD, Nguyen HTT, Gammeltoft T, et al. Intimate partner violence during pregnancy in Vietnam: prevalence, risk factors and the role of social support. Global Health Action. 2018;11(sup3):1638052.

5. Kana MA, Safiyan H, Yusuf HE, Musa ASM, Richards-Barber M, Harmon QE, et al. Association of intimate partner violence during pregnancy and birth weight among term births: a cross-sectional study in Kaduna, Northwestern Nigeria. BMJ Open. 2020;10(12):e036320.

6. Teshome A, Gudu W, Bekele D, Asfaw M, Enyew R, Compton SD. Intimate partner violence among prenatal care attendees amidst the COVID-19 crisis: The incidence in Ethiopia. International Journal of Gynecology & Obstetrics. 2021;153(1):45-50.

7. Manongi R, Rogathi J, Sigalla G, Mushi D, Rasch V, Gammeltoft T, et al. The Association Between Intimate Partner Violence and Signs of Depression During Pregnancy in Kilimanjaro Region, Northern Tanzania. Journal of Interpersonal Violence. 2017;35(23-24):5797-811.

8. Schneider M, Baron E, Davies T, Munodawafa M, Lund C. Patterns of intimate partner violence among perinatal women with depression symptoms in Khayelitsha, South Africa: a longitudinal analysis. Glob Ment Health (Camb). 2018;5:e13-e.

9. Gashaw BT, Schei B, Magnus JH. Social ecological factors and intimate partner violence in pregnancy. PloS one. 2018;13(3):e0194681-e.

10. Tesfaye Y, Agenagnew L. Antenatal Depression and Associated Factors among Pregnant Women Attending Antenatal Care Service in Kochi Health Center, Jimma Town, Ethiopia. Journal of Pregnancy. 2021;2021:5047432.

11. Ezeudu CC, Akpa O, Waziri NE, Oladimeji A, Adedire E, Saude I, et al. Prevalence and correlates of intimate partner violence, before and during pregnancy among attendees of maternal and child health services, Enugu, Nigeria: mixed method approach, January 2015. Pan Afr Med J. 2019;32(Suppl 1):14-.

12. Nasreen HE, Rahman JA, Rus RM, Kartiwi M, Sutan R, Edhborg M. Prevalence and determinants of antepartum depressive and anxiety symptoms in expectant mothers and fathers: results from a perinatal psychiatric morbidity cohort study in the east and west coasts of Malaysia. BMC Psychiatry. 2018;18(1):195-.

13. Shrestha M, Shrestha S, Shrestha B. Domestic violence among antenatal attendees in a Kathmandu hospital and its associated factors: a cross-sectional study. BMC Pregnancy Childbirth. 2016;16(1):360-.

14. Khatoon F, Fatima M, Zaidi Z, Nishad S, Ahmad A. Domestic Violence During Pregnancy: Evaluating the Impact on Maternal and Perinatal Health—A Pilot Study in Uttar Pradesh. The Journal of Obstetrics and Gynecology of India. 2021.

15. Naghizadeh S, Mirghafourvand M, Mohammadirad R. Domestic violence and its relationship with quality of life in pregnant women during the outbreak of COVID-19 disease. BMC Pregnancy Childbirth. 2021;21(1):88.

16. McKelvie S, Leodoro B, Sala T, Tran T, Fisher J. Prevalence, Patterns, and Determinants of Intimate Partner Violence Experienced by Women Who Are Pregnant in Sanma Province, Vanuatu. Journal of Interpersonal Violence. 2020:0886260520969235.

17. Lobato G, Reichenheim ME, Moraes CL, Peixoto-Filho FM, Migowski LS. Psychologic intimate partner violence and the risk of intrauterine growth restriction in Rio de Janeiro. International Journal of Gynecology & Obstetrics. 2018;143(1):77-83.

18. Ribeiro MRC, Batista RFL, Schraiber LB, Pinheiro FS, Santos AMd, Simões VMF, et al. Recurrent Violence, Violence with Complications, and Intimate Partner Violence Against Pregnant Women and Breastfeeding Duration. Journal of Women's Health. 2020.

19. Navarrete L, Nieto L, Lara MA. Intimate partner violence and perinatal depression and anxiety: Social support as moderator among Mexican women. Sexual & Reproductive Healthcare. 2021;27:100569.

20. Laelago T, Belachew T, Tamrat M. Effect of intimate partner violence on birth outcomes. Afr Health Sci. 2017;17(3):681-9.

21. le Roux K, Christodoulou J, Stansert-Katzen L, Dippenaar E, Laurenzi C, le Roux IM, et al. A longitudinal cohort study of rural adolescent vs adult South African mothers and their children from birth to 24 months. BMC Pregnancy Childbirth. 2019;19(1):24-.

22. Luhumyo L, Mwaliko E, Tonui P, Getanda A, Hann K. The magnitude of intimate partner violence during pregnancy in Eldoret, Kenya: exigency for policy action. Health Policy and Planning. 2020;35(Supplement_1):i7-i18.

23. Jain S, Varshney K, Vaid NB, Guleria K, Vaid K, Sharma N. A hospital-based study of intimate partner violence during pregnancy. Int J Gynaecol Obstet. 2017;137(1):8-13.

24. Kashanian M, Faghankhani M, YousefzadehRoshan M, EhsaniPour M, Sheikhansari N. Woman’s perceived stress during pregnancy; stressors and pregnancy adverse outcomes. The Journal of Maternal-Fetal & Neonatal Medicine. 2021;34(2):207-15.

25. Afkhamzadeh A, Rahmani K, Yaghubi R, Ghadrdan M, Faraji O. Adverse perinatal outcomes of intimate partner violence during pregnancy. International Journal of Human Rights in Healthcare. 2021;ahead-of-print(ahead-of-print).
